# Factor-GAN: Enhancing stock price prediction and factor investment with Generative Adversarial Networks

**DOI:** 10.1371/journal.pone.0306094

**Published:** 2024-06-25

**Authors:** Jiawei Wang, Zhen Chen

**Affiliations:** 1 School of Finance, Shanghai University of Finance and Economics, Shanghai, China; 2 School of Electronic Information and Electrical Engineering, Shanghai Jiao Tong University, Shanghai, China; Beihang University, CHINA

## Abstract

Deep learning, a pivotal branch of artificial intelligence, has increasingly influenced the financial domain with its advanced data processing capabilities. This paper introduces Factor-GAN, an innovative framework that utilizes Generative Adversarial Networks (GAN) technology for factor investing. Leveraging a comprehensive factor database comprising 70 firm characteristics, Factor-GAN integrates deep learning techniques with the multi-factor pricing model, thereby elevating the precision and stability of investment strategies. To explain the economic mechanisms underlying deep learning, we conduct a subsample analysis of the Chinese stock market. The findings reveal that the deep learning-based pricing model significantly enhances return prediction accuracy and factor investment performance in comparison to linear models. Particularly noteworthy is the superior performance of the long-short portfolio under Factor-GAN, demonstrating an annualized return of 23.52% with a Sharpe ratio of 1.29. During the transition from state-owned enterprises (SOEs) to non-SOEs, our study discerns shifts in factor importance, with liquidity and volatility gaining significance while fundamental indicators diminish. Additionally, A-share listed companies display a heightened emphasis on momentum and growth indicators relative to their dual-listed counterparts. This research holds profound implications for the expansion of explainable artificial intelligence research and the exploration of financial technology applications.

## 1 Introduction

In contemporary financial research, the primary objective of asset pricing models is to identify the key factors influencing market returns [1]. Traditionally, multi-factor pricing models have been extensively employed to elucidate stock market anomalies [2]. However, the surge of big data has introduced considerable noise and uncertainty into the financial market. As the dimensions of factors increase, the complexity of the prediction function sharply rises, rendering traditional methods less suitable for analyzing the intricate, high-dimensional, and noisy data series prevalent in the financial market [3, 4]. Facing these challenges, machine learning demonstrates a natural aptitude for processing unstructured data and extracting potential features embedded in information [5]. Therefore, incorporating machine learning techniques into multi-factor pricing models is crucial for enhancing the predictability of asset pricing factors.

Deep learning (DL), an advanced machine learning technology based on artificial neural networks, stands as a promising branch that has garnered attention from researchers [6, 7]. Compared with traditional algorithms, deep learning exhibits higher accuracy and generalization ability in recognition and classification tasks. The essence of deep learning lies in its automatic extraction of features from data at each layer through the learning process, eliminating the need for artificial feature selection [8]. This technology has showcased achievements across various domains, including image processing, text mining, speech recognition, and natural language processing [9, 10]. Notably, in recent applications, it has been instrumental in revolutionizing areas such as autonomous vehicles [11], healthcare diagnostics [12], and financial technology [13], demonstrating its adaptability and efficacy in addressing real-world challenges.

A pivotal application of deep learning in the financial domain is stock price prediction [14, 15], where investors seek robust algorithms to navigate extremely noisy and volatile markets. Deep learning methods can rapidly process extensive and intricate datasets, representing nonlinear functions without being constrained by dimensionality. This characteristic helps investors extract valuable insights and make well-informed decisions in complex market scenarios [16]. Our study is at the forefront of applying deep learning techniques to stock return prediction and factor investment. We utilize deep learning methods for data reduction and feature extraction, addressing the challenge of high-dimensional data traps. By doing so, we efficiently uncover both observable and hidden information within stock characteristics. Furthermore, unlike linear regression methods, deep learning takes into account the nonlinear dependency within data. This allows us to effectively capture nonlinear information within the extensive datasets.

The Generative Adversarial Network (GAN) is a machine learning architecture comprising a generator and a discriminator [17, 18]. In this setup, the generator simulates data akin to real data, acting as a “cheater”, while the discriminator judges real and generated data. Through confrontation, they converge to a point where the discriminator cannot differentiate between the two types of data. Since its birth, GAN has experienced rapid development, with leading tech companies investing in its applications including computer vision, signal processing, and image synthesis [19, 20]. However, few studies have explored GAN networks in the realm of asset pricing. Existing literature often focuses on mature European and American capital markets, examining only a handful of specific stocks, thereby limiting the generalizability of conclusions [21, 22]. China’s stock market, marked by a significant retail investor presence, government information influence, and high market volatility, presents a unique landscape [23, 24]. This study marks, to the best of our knowledge, the first application of GAN in factor selection for predicting stock returns. Furthermore, it represents the pioneering exploration of applying deep learning and asset pricing to China’s emerging stock market.

In this study, we present an innovative asset pricing model that integrates deep neural networks with the multi-factor model. Specifically, we introduce Factor-GAN, an intelligent stock price prediction framework that leverages Generative Adversarial Networks (GAN) for return forecasting and factor investing. Factor-GAN employs Long Short-Term Memory (LSTM) as a generator and Convolutional Neural Network (CNN) as a discriminator, optimizing parameters through a zero-sum game mechanism. Furthermore, to enhance the interpretability of deep learning methods, we conduct a subsample analysis aimed at exploring decisive factors across various categories of companies.

The theoretical and practical contributions of this paper are as follows:

- This study pioneers the integration of deep learning technology with multi-factor pricing models, contributing to the expansion of existing asset pricing literature. Traditional asset pricing models, which predominantly rely on linear regressions, often fail to capture the complexity, sparsity, and non-linearity inherent in financial datasets. By constructing a factor database that encapsulates firm-level characteristics, our study utilizes deep learning to extract and analyze stock factor information, leading to notable enhancements in prediction accuracy and model stability.
- We introduce Factor-GAN, a cutting-edge forecasting framework that applies GANs to the realms of stock return prediction and factor investing. Factor-GAN adopts a “zero-sum game” mechanism, utilizing LSTM networks for processing financial time series data and one-dimensional CNN for classification tasks. This approach aims to refine the predictive model until it can generate data indistinguishable from real observations by the discriminator. Empirical evidence supports the superior performance of Factor-GAN, which yields an annualized return of 23.52% and a Sharpe ratio of 1.29.
- This research contributes to the domain of explainable artificial intelligence by investigating the economic principles underpinning deep learning applications in finance. Through a targeted subsample analysis within the unique context of China’s stock market, we reveal that during the transition from SOEs to non-SOEs, fundamental factors diminish in variable importance, making way for the significance of liquidity and volatility indicators. A-share listed companies pay more attention to momentum and growth indicators compared to dual-listed counterparts.

The remainder of the paper is structured as follows. Section 2 reviews the literature on deep learning techniques for asset pricing. Section 3 illustrates the dataset and the proposed Factor-GAN architecture and its underlying mathematical model. Section 4 presents results of experiments with all stocks from the Chinese A-share market. Section 5 makes a subsample analysis of the nature of enterprises and the place of listing. Section 6 concludes the paper and provides directions for future scopes of the research.

## 2 Related literature

### 2.1 Multi-factor asset pricing models

The Capital Asset Pricing Model (CAPM) serves as the foundational framework for multi-factor models [25]; however, empirical evidence indicates that market risk alone cannot comprehensively explain stock returns. Fama and French [26] introduced a three-factor model (FF3), extending CAPM with scale and market-to-book ratio factors. Although FF3 enhanced our understanding of average returns, it encountered challenges. Notably, Carhart [27] addressed the momentum effect by proposing a four-factor model that incorporated a momentum factor. Despite these advancements, FF3 faced limitations in explaining the relationship between corporate investment styles and cross-sectional returns. Aharoni et al. [28] found that higher investment expenditure correlated with lower future stock returns. Furthermore, FF3 failed to capture the negative relationship between corporate operating profits and stock prices [29]. Recognizing these empirical deficiencies, Fama and French [30] introduced the five-factor model, augmenting FF3 with profitability and investment factors, significantly improving its explanatory power for cross-sectional returns.

In the realm of multi-factor pricing models, machine learning methods have emerged as powerful tools for extracting patterns from extensive datasets, opening new avenues for innovative research. For instance, Bianchi et al. [31] demonstrated the efficacy of neural network algorithms in predicting bond excess returns using macroeconomic information. Li et al. [32] applied representative machine learning algorithms to construct a factor pricing model for China’s A-share market, revealing their effectiveness in identifying abnormal factors and predicting stock price trends. Chen and Ge [33] enhanced LSTM networks by incorporating an attention mechanism. Their model, based on 18 daily technical indicators, successfully predicted the direction of share prices in the Hong Kong stock market.Yıldırım et al. [34] proposed a hybrid RNN model, addressing macroeconomic forecasting indicators and technical indicators separately. The hybrid framework demonstrated superior performance in predicting daily volatility in the foreign exchange market compared to linear methods. These studies underscore the evolving of multi-factor asset pricing models, with machine learning playing a pivotal role in enhancing predictive accuracy and capturing complex relationships in financial markets.

### 2.2 Application of deep learning in asset pricing

Deep learning, a promising branch of machine learning, has found extensive application in diverse domains such as speech recognition, image classification, and language processing. However, its exploration in the financial sector remains in its young stages [35, 36]. Providing a comprehensive overview, Li and Ma [37] conducted a survey on the application of neural networks in forecasting financial market prices. This encompassed predictions related to stock prices, option pricing, exchange rates, as well as banking and financial crises. In a pioneering effort, Gu et al. [38] conducted a comparative analysis of machine learning methods, including multilayer neural networks, for predicting U.S. stock returns. Their work underscored the advantages of employing flexible forms of nonlinear functions.

Recent scholarly attention has shifted towards the utilization of deep neural networks (DNN) in asset pricing. Krausa and Feuerriegel [39] investigated the use of DNN for financial decision support. Their research unveiled higher directional accuracy in DNN compared to traditional machine learning methods and the RNN model when predicting stock price movements in response to financial disclosures. Chen et al. [40] established a DNN-based stock index futures prediction model incorporating autoencoders and restricted Boltzmann machines. Analyzing empirical data from the CSI300 futures contract at high frequency, they found that the deep learning method surpassed backpropagation in both fitting degree and directional predictive accuracy. Feng et al. [41] introduced a no-arbitrage constraint by employing a set of pre-specified linear asset pricing factors, estimating risk loadings with DNN. Meanwhile, Zheng et al. [42] focused on exchange rate forecasting using a deep belief network (DBN). Experimental results demonstrated that the improved DBN model outperformed traditional machine learning models, with a smaller number of layer nodes yielding a more significant impact.

Predicting stock market movements has been a classic challenge, but deep learning techniques have recently made notable progress in forecasting stock prices. Nelson et al. [43] explored the application of LSTM networks in predicting future stock price trends. Leveraging price history and technical analysis indicators, their results exhibited promise, achieving an average accuracy of 55.9%. Selvin et al. [44] delved into the architecture of CNN-sliding window to predict stock index movements. Contrary to existing non-linear algorithms, CNN demonstrated the ability to capture dynamic changes in data within India-listed companies. Li and Tan [45] proposed a Deep Rank network for ranking stocks based on excess returns, establishing a comprehensive procedure for classification, ranking, and neural network scoring. Hiransha et al. [46] examined four deep learning models, including CNN, LSTM, RNN, and MLP, for stock price prediction, revealing the superior performance of deep learning models over non-linear models like ARIMA.

The emergence of hybrid systems combining deep learning techniques has also been noteworthy. Babu and Reddy [47] integrated the ARIMA model with neural networks to predict financial time-series. Results demonstrated that the hybrid model exhibited higher prediction accuracy for both one-step-ahead and multi-step-ahead forecasts, surpassing the individual ARIMA and NN models. Matsubara et al. [48] harnessed sentiment information from news articles, constructing a hybrid model combining DNN and a generative model for stock price movement prediction. Jiang et al. [49] proposed a cross-domain deep learning approach, Cd-DLA, to capture time-series interactions in financial data for multiple stock market predictions, outperforming simple machine learning methods in currency markets. In a recent development, Dezhkam and Manzuri [50] introduced the HHT-XGB model, employing Hilbert-Huang Transform (HHT) for feature engineering and XGBoost for classifying closing price trends. The model generates a sequence of ups and downs, optimizing stock portfolio weights for enhanced trading performance.

Our paper contributes to the expanding research on asset pricing by employing deep learning methods, especially given the limited achievements in using GAN models in finance thus far. Originally proposed by Goodfellow et al. [7] for image recognition, the GAN model has recently gained traction in finance. Chen et al. [51] pioneered the use of GAN to construct a nonlinear asset pricing model with a substantial amount of conditioning information. Their approach incorporated a feedforward network for the generator and an RNN model for the discriminator. The experimental results demonstrated that GAN-based portfolio returns and Sharpe ratios significantly surpassed those of the Fama-French model in the U.S. market. Diqi et al. [52] introduced Stock-GAN, leveraging the GAN algorithm for robust stock price prediction. Their model utilized features such as date, open, high, low, close, and volume of stocks in Indonesia market. The experiments yielded high accuracy and a low error rate, suggesting Stock-GAN as a promising solution for accurate and dynamic stock price predictions. Li et al. [53] introduced a new method for predicting stock prices using GANs, focusing on analyzing stock text. They aimed to improve the accuracy of stock text classification by refining the text emotion classification model. The results showed that their approach outperforms traditional models, emphasizing the effectiveness of integrating GANs with text analysis for stock price prediction. Vuletić et al. [54] explored the GAN model for predicting financial time series. They developed a new loss function for GANs, improving their performance in classification tasks. By generating probability distributions of price returns based on historical data, the GAN-based approach achieved higher accuracy compared to traditional models like LSTMs and ARIMA. Zhang et al. [55] proposed a GAN architecture with the MLP as the discriminator and the LSTM as the generator for forecasting the S&P 500 Index. The experimental outcomes showcased excellent performance in closing price prediction on real data compared to other deep learning models.

Our study represents a pioneering effort as the first to apply the GAN framework to factor selection for predicting future stock returns. Simultaneously, our research breaks new ground by applying deep learning techniques to the Chinese stock market, an emerging market with unique characteristics.

## 3 Data and methodology

**Fig 1:**
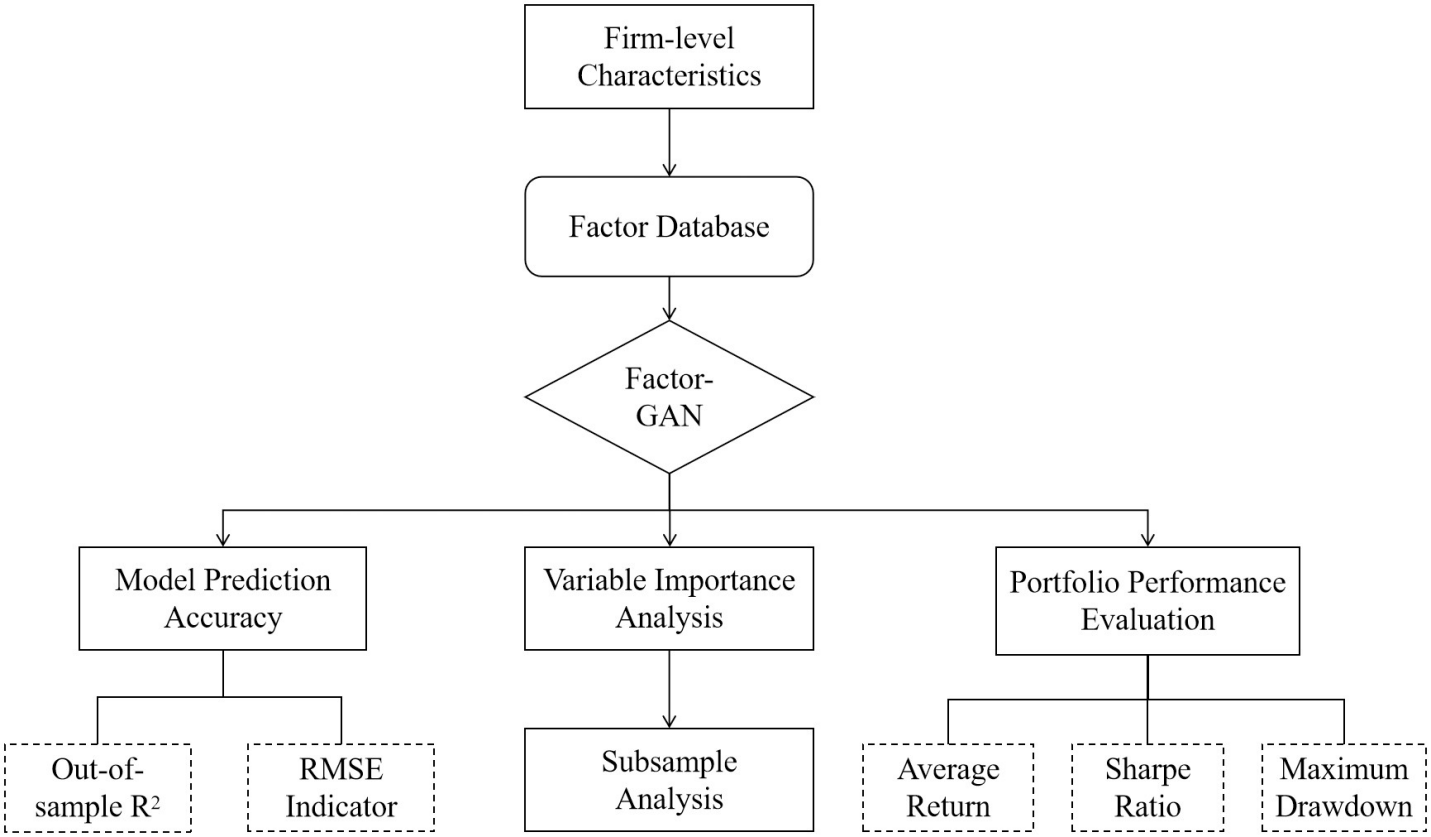
The flowchart of research framework.

In this section, we begin by providing details of the experimental dataset, followed by a description of the multi-factor asset pricing model based on deep learning. Subsequently, we introduce Factor-GAN, an innovative application of GAN models for forecasting stock returns and factor investing. For a visual representation of our research framework, please refer to Fig 1.

### 3.1 Dataset

The data for this paper is sourced from the Wind platform. We collect the return data of all stocks in the Chinese A-share market spanning 19 years, from January 2002 to December 2020. The risk-free rate is represented by the monthly yield of one-year treasury bonds. Market portfolio returns are calculated as the weighted average of the returns of all stocks in the Chinese A-share market, weighted by circulating market capitalization. To ensure the stability of results, this study excludes stocks with special treatment, less than one year of listing, and those delisted midway. Finally, the total sample for the study includes 3619 stocks.

Following the research methods of Leippoid et al. [56] and Wang et al. [57], we establish a large-scale firm-level factor database. These factors encompass 70 dimensions, comprehensively measuring the overall operations of firms, and are classified into 9 different groups: Beta, Valuation, Profitability, Growth, Leverage, Liquidity, Momentum, Size, and Volatility. Additionally, this paper introduces two binary variables to represent the ownership form and listing method of stocks, denoted as *soe* and *dual* − *listing*, respectively. For detailed definitions and classifications of the factors, please refer to the S1 Appendix.

To ensure the comparability of factor contributions, considering the different magnitudes among variables, we standardize each feature. Inspired by the research of Gu et al. [38], we rank all stock features cross-sectionally for each period and map these rankings to the [-1,1] interval. For basic statistical descriptions of the factors, please refer to the S1 Appendix.

### 3.2 Asset pricing model based on deep learning

This paper innovatively integrates a traditional multi-factor pricing model from asset pricing fields with deep learning techniques, resulting in a powerful deep learning-based multi-factor pricing model:

$$\begin{array}{c} r_{s,t}=E_{t}(r_{s,t-1})+\mu_{s,t} \end{array}$$ (1)

$$\begin{array}{c} E_{t}(r_{s,t-1})=f_{t}^{*}(z_{s,t-1};\theta ) \end{array}$$ (2)

where *r*_*s*,*t*_ represents the return of stock *s* in period *t*, *E*_*t*_(*r*_*s*,*t*−1_) is the expected return in period *t*, *μ*_*s*,*t*_ denotes the residual term. The function $f_{t}^{*}(z_{s,t-1};\theta )$ encompasses various nonlinear function models for period *t*, and *z*_*s*,*t*−1_ corresponds to the firm characteristics of stock *s* in period *t* − 1.

The combination of deep learning and the multi-factor pricing model offers several advantages. Firstly, the robust data processing abilities of deep learning networks improve the accuracy of traditional multi-factor models in predicting stock prices. Deep learning excels at capturing intricate patterns, nonlinear relationships, and evolving dependencies within financial data, making it well-suited for addressing pricing issues in finance domain. Moreover, the multi-factor pricing model based on deep learning considers a large-scale of firm-level factors. By employing deep networks to extract and analyze latent information within factors, it overcomes the constraints of linear models in handling high-dimensional data and addresses the challenge of factor selection in dynamic market environments.

Consistent with the approaches of Gu et al. [38], we employ the expanding windows method to divide our dataset into training, validation, and testing sets. Initially, the training set spans from January 2002 to December 2010, followed by the validation set covering January 2011 to December 2013. The resulting forecasting model then estimates stock returns for the sample period, extending from January 2014 to December 2014. Subsequently, at the beginning of each year, the lengths of the validation and test sets remain constant, while the training set extends by one additional year.

### 3.3 GAN networks for stock return prediction

#### 3.3.1 LSTM

Recurrent Neural Networks (RNN) represent a class of neural networks adept at handling sequence-to-sequence tasks. This allows RNNs to preserve information from prior inputs, making them particularly suited for learning dependencies within sequential data. Despite their strengths, RNNs encounter difficulties with longer sequences, grappling with challenges like vanishing and exploding gradients that can hinder learning over extended periods.

In response to these limitations, Long Short-Term Memory (LSTM) networks were developed as an advanced iteration of RNN. Introduced by Hochreiter and Schmidhuber in 1997, LSTMs have garnered acclaim for their efficacy, particularly in applications requiring the analysis of data sequences [36]. Distinguished from traditional RNNs, LSTMs excel in capturing long-term dependencies within time series data, making them ideally suited for the complex task of stock price prediction.

**Fig 2:**
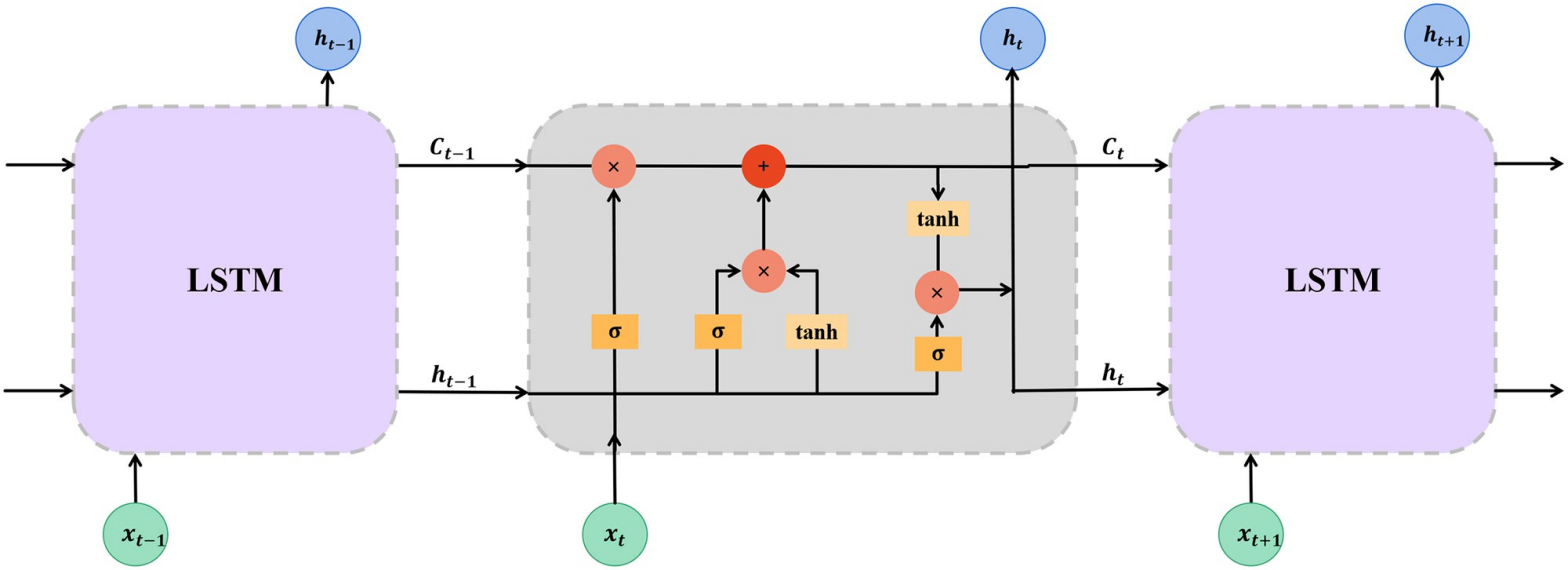
The architecture of the LSTM model.

At the core of LSTM is the memory cell, a dynamic element allowing the network to retain information across multiple time steps. This is complemented by three specialized gates: input, forget, and output, which collectively manage the cell’s information flow. These gates permit the LSTM to add, discard, or pass through information, ensuring that only relevant data influences the prediction. By leveraging these capabilities, LSTMs offer a powerful tool for forecasting stock prices, adept at analyzing financial time series data and identifying meaningful patterns that precede significant market movements. Fig 2 depicts the structure of a typical LSTM network.

The mathematical formulation for the LSTM model, as applied to stock price prediction, is outlined below. In this context, each input *x*_*t*_ represents the stock factors of each stock at time *t*, and the output aims to predict the stock price:

$$\begin{array}{c} \left\{ \begin{array}{c} f_{t}=\sigma (W_{f}x_{t}+b_{f}+U_{f}h_{t-1}+b_{f}^{\prime })\\ i_{t}=\sigma (W_{i}x_{t}+b_{i}+U_{i}h_{t-1}+b_{i}^{\prime })\\ g_{t}=\text{tanh}(W_{g}x_{t}+b_{g}+U_{g}h_{t-1}+b_{g}^{\prime })\\ o_{t}=\sigma (W_{o}x_{t}+b_{o}+U_{o}h_{t-1}+b_{o}^{\prime })\\ c_{t}=f_{t}⊙c_{t-1}+i_{t}⊙g_{t}\\ h_{t}=o_{t}⊙\text{tanh}(c_{t}) \end{array}\right. \end{array}$$ (3)

where *h*_*t*_ is the hidden state, *c*_*t*_ is the cell state, crucial for capturing temporal dependencies. The model inputs, *x*_*t*_, consist of a vector of stock factors for each stock at time *t*, serving as the basis for prediction. The gates within the LSTM—forget gate (*f*_*t*_), input gate (*i*_*t*_), and output gate (*o*_*t*_), alongside the cell update gate (*g*_*t*_)—manage the information flow through the network.

The weights W = {W_f_, W_i_, W_g_, W_o_} and biases $B=\{b_{f},b_{i},b_{g},b_{o},b_{f}^{\prime },b_{i}^{\prime },b_{g}^{\prime },b_{o}^{\prime }\}$ are parameters learned during training. The sigmoid function, *σ*, controls the gate activation. The Hadamard product, ⊙, facilitates element-wise multiplication, ensuring a dynamic update of the cell state. And the hidden state, *h*_*t*_, based on the input stock factors. This formulation allows the LSTM to capture complex, non-linear relationships between the stock factors and their subsequent effect on stock price predictions.

#### 3.3.2 The architecture of GAN

Predicting stock price movements using market data poses a challenging task. In 2014, Ian Goodfellow introduced a machine learning framework for GAN in his seminal work ‘Generative Adversarial Nets’ [7]. GAN operates within a game-theory setting where two neural networks engage in a competitive interplay and excels in learning to generate new data that closely resembles the training set [35]. This capability makes them particularly well-suited for the complex and dynamic nature of stock market data.

**Fig 3:**
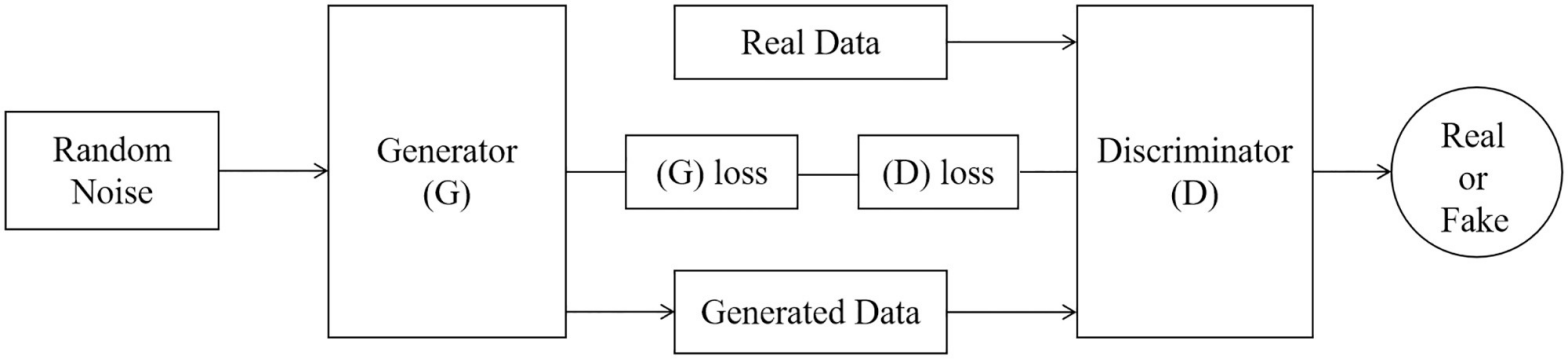
The architecture of typical GAN networks.

The GAN framework comprises two essential components: the generator(G) and the discriminator(D). The generator network creates data samples that closely resemble real data, while the discriminator network assesses whether a given sample originates from the real data or the generator. Fig 3 illustrates the architecture of GAN networks.

The training process is essentially a zero-sum game between these two entities. Given the probability distribution of samples *P*_*data*(*x*)_, our objective is to train *G* and *D* so that *P*_*G*(*x*)_ = *P*_*data*(*x*)_, rendering *D* incapable of distinguishing between the two. The objective function of GAN is as follows:

$$\begin{array}{c} \underset{G}{\text{min}}\;\underset{D}{\text{max}}\;V\left(D,G\right)=E_{x∼P\text{data}(x)}\left[\text{log}\;D\left(x\right)\right]+E_{z∼Pz(z)}\left[\text{log}\left(1-D\left(G\left(z\right)\right)\right)\right] \end{array}$$ (4)

where *x* represents the real data, and *z* stands for the input noise utilized by the Generator. The outputs of the discriminator and the generator are denoted as *D*(*x*) and *G*(*z*), respectively. The variable *D*(*x*) takes on scalar values of 0 or 1, signifying whether it classifies a sample as real or fake. In the context of our training dataset, *P*_*data*(*x*)_ signifies the probability distribution of samples derived from real data. *P*_*Z*(*z*)_ represents the probability distribution of samples generated by the noise generator.

The performance of GAN is heavily influenced by data structure and model complexity. When the discriminator becomes overly accurate, the update of generator parameters may struggle to converge. To address the issues of gradient vanishing and model collapse, subsequent researchers introduced the Wasserstein GAN (WGAN). WGAN replaces the Jensen-Shannon (JS) divergence with the Wasserstein distance as the metric for quantifying the dissimilarity between two distributions. This modification imparts WGAN with favorable convergence properties, leading to a more stable model optimization process, making it well-suited for applications like stock price prediction.

The formula of the WGAN for stock price prediction is as follows:

$$\begin{array}{c} L=E_{x∼\text{Pdata}(r_{s,t})}[f_{w}(r_{s,t})]-E_{z∼Pz(z_{s,t-1})}[f_{w}(G(z_{s,t-1}))] \end{array}$$ (5)

where *r*_*s*,*t*_ represents the actual return of stock *s* at time *t*, and *G*(*z*_*s*,*t*−1_) is the predicted return. *z*_*s*,*t*−1_ signifies the firm characteristic factor of stock *s* at time *t* − 1. *f*_*w*_ denotes the new discriminator, and *L* represents the discrepancy between the estimated and actual values. The generator is optimized iteratively to minimize *L*, such that the estimated return ${\hat{r}}_{s,t,G}$ when *L* reaches its minimum point, ultimately serving as our model’s output.

#### 3.3.3 Factor-GAN

The characteristics of GAN models enable their application in financial asset pricing. Unlike traditional neural network models relying on back-propagation for parameter updates, GANs introduce an adversarial network system. The generator uses factor datasets to predict stock market returns, while the discriminator compares real and predicted returns to guide the generator in updating parameters. Throughout the iteration process, both parties continuously enhance their abilities to reach Nash equilibrium. Therefore, the final prediction results are closer to the actual return distribution compared to single neural network models.

Previous studies have predominantly focused on using historical stock prices to predict future returns, neglecting the wealth of information embedded in high-dimensional factors. This paper constructs an innovative framework called Factor-GAN, employing the GAN model to factor selection process to enhance stock price prediction. In Factor-GAN, LSTM serves as the generator and CNN as the discriminator. On the one hand, LSTM excels in capturing long-term dependencies from time series, offering a natural advantage in analyzing the impact of factors on stock prices. On the other hand, the CNN model is adept at handling spatial data and feature detection, making it well-suited for classification tasks and discerning real returns from predicted returns.

**Fig 4:**
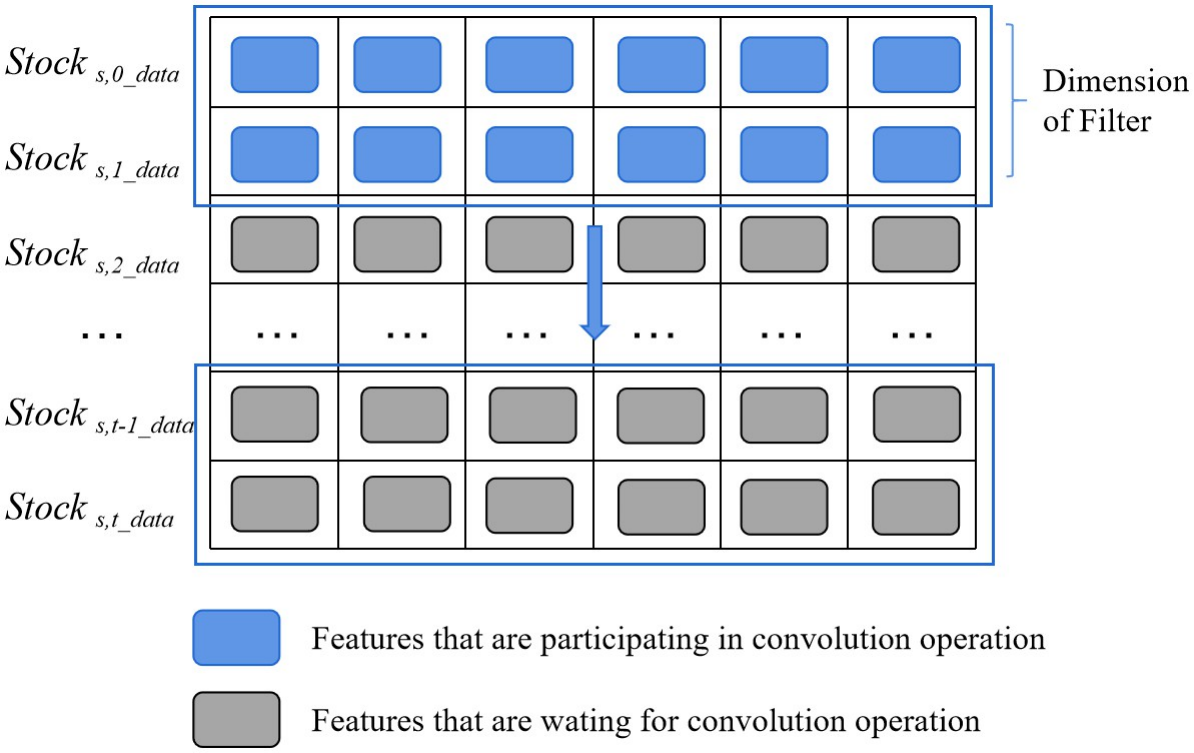
The architecture of one-dimensional CNN.

Specifically, we choose a one-dimensional CNN. Unlike two-dimensional CNNs, one-dimensional CNNs perform convolution operations in a single dimension, making them a fitting choice for sequential models, such as time series prediction and natural language processing. In the scenario of stock prediction, the one-dimensional convolutional structure is depicted in Fig 4, where each row represents a time dimension and each column corresponds to a firm characteristic.

In this study, the parameter settings for the GAN model are determined through a series of experiments. For the LSTM component, we utilize 70 input units and 500 hidden units, employing the LeakyReLu activation function. Our CNN architecture is constructed with a three-layer one-dimensional convolutional layer and a two-layer fully connected layer. Each convolutional layer consists of 32, 64, and 128 convolution kernels, with a *kernel*_*size* set at 5. The fully connected layer comprises 220 neurons. Additionally, we set the model to iterate 200 times, use *batch*_*size* of 64, the *timestep* of 1, the dropout parameter to 0.1, and establish a learning rate of 0.001. Our choice of optimizer is Adam, which dynamically adjusts the learning rate of model parameters, resulting in enhanced sample data quality and expedited model convergence. The GAN model is optimized using the stochastic gradient descent (SGD) iteration method.

**Fig 5:**
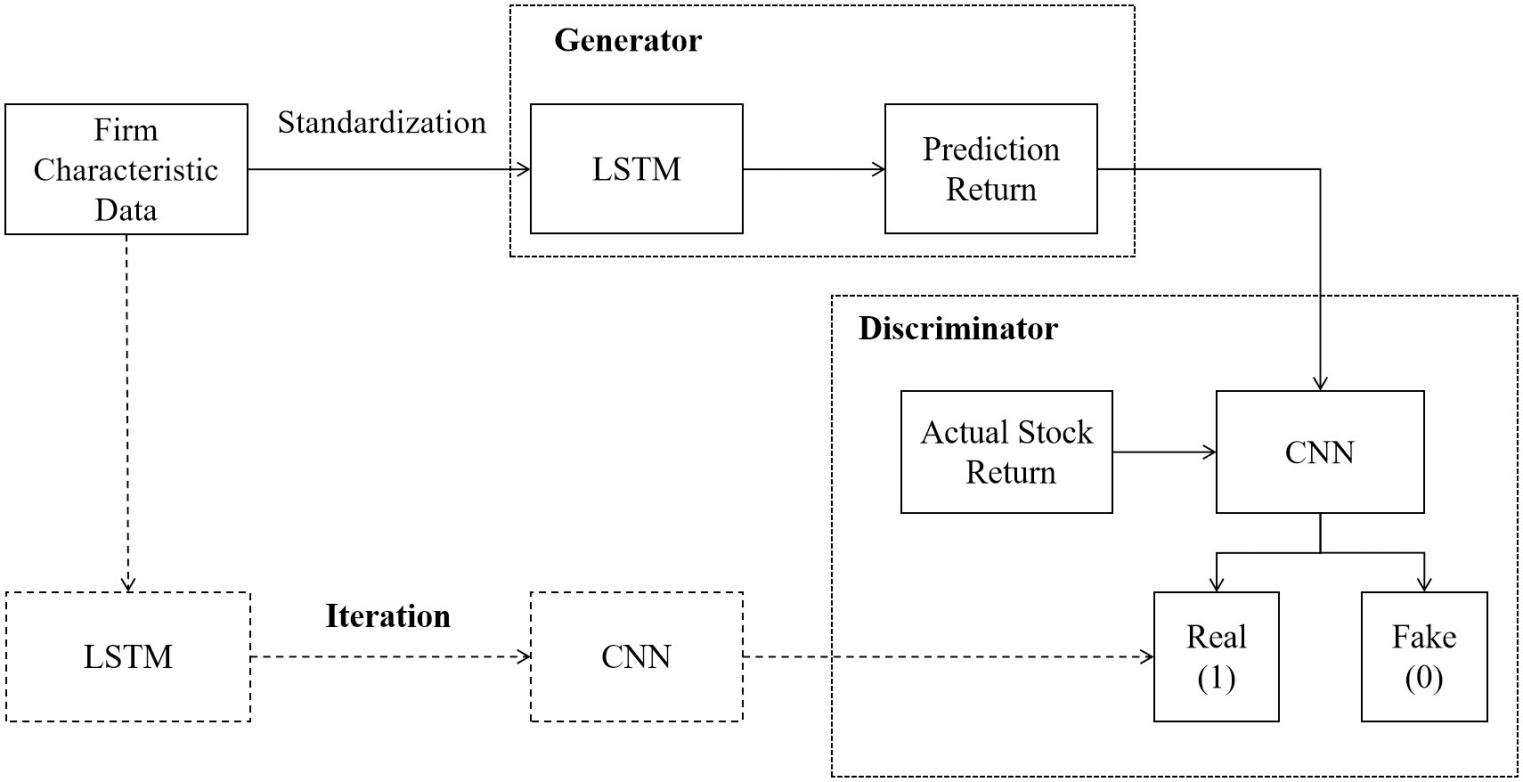
The process of using Factor-GAN for stock price prediction.

The process of predicting stock returns with Factor-GAN involves several key steps, as illustrated in Fig 5.

1. Normalization of Firm Characteristic: Standardize the firm characteristic data for individual stock and utilize it as input for training the generator to obtain initial predicted values.
2. Discrimination of Predicted Values: Utilize the discriminator to discern between the generated predicted values, categorized as ‘0’, and the actual stock returns, marked as ‘1’.
3. Parameter Locking and Iterative Generation: Following a training cycle, lock the parameters of the discriminator. Engage in an iterative process of generating new predicted values with the generator, classifying these values as ‘1’ to the greatest extent possible.
4. Overfitting Mitigation: Introduce a validation set and halt training when the loss function for the validation set no longer decreases. Separately, employ this generator model to forecast stock returns for period *t* + 1.
5. Iterative Completion: Execute the above steps iteratively to accomplish the prediction process for all out-of-sample periods.

### 3.4 Performance evaluation

In assessing the performance of stock return forecasts, we follow the approach commonly used in the previous literature [38, 58]. To accurately measure the predictive effects of various methods, we rely on the non-demeaned out-of-sample *R*^2^ to have a direct comparison:

$$\begin{array}{c} R_{\text{os}}^{2}=1-\frac{\sum_{(s,t)\in T}{\left(R_{s,t}-{\hat{R}}_{s,t}\right)}^{2}}{\sum_{(s,t)\in T}R_{s,t}^{2}} \end{array}$$ (6)

where *R*_*s*,*t*_ denotes the return of stock *s* at time *t*, ${\hat{R}}_{s,t}$ are predicted monthly return of stock *s* at time *t*. *T* denotes the set of predictions that are only assessed on the testing sample, in other words, the data never enter into model estimation or tuning. The $R_{\text{os}}^{2}$ ranges from (−∞, 1], where higher values indicate better forecasting performance of the model.

In addition, following Lin et al. [17], we also evaluated the performance of each model by Root Mean Square Error (RMSE). The RMSE is used to measure the forecasting accuracy of the forecasting model on continuous data and represents the average degree of deviation between the predicted value and the actual value. The indicator is defined as:

$$\begin{array}{c} RMSE=\sqrt{\frac{1}{T}\frac{1}{N}\sum_{t=1}^{T}\sum_{s=1}^{N}(r_{s,t}-{{\hat{r}}_{s,t}}^{2})} \end{array}$$ (7)

In the realm of factor investing, evaluating the performance and risk of investment strategies is crucial. Four key indicators are commonly used to gauge these aspects: Average Predicted Monthly Return (Avg), Standard Deviation of Monthly Returns (Std), Sharpe Ratio (SR), and Maximum Drawdown (MaxDD). The indicators are as follows:

$$\begin{array}{c} Avg=\frac{1}{N}\sum_{i=1}^{N}R_{i} \end{array}$$ (8)

$$\begin{array}{c} Std=\sqrt{\frac{1}{N-1}\sum_{i=1}^{N}{\left(R_{i}-Avg\right)}^{2}} \end{array}$$ (9)

$$\begin{array}{c} SR=\frac{Avg-R_{f}}{Std} \end{array}$$ (10)

$$\begin{array}{c} MaxDD=\underset{t\in [0,T]}{\text{max}}(\underset{t^{\prime }\in \left[0,t\right]}{\text{max}}(V_{t^{\prime }}-V_{t})) \end{array}$$ (11)

where *R*_*i*_ is the return in month *i*, *N* is the total number of months, and *R*_*f*_ is the risk-free rate. *V*_*t*_ is the portfolio value at time *t*, and *T* is the total observation period.

## 4 Experiment results

### 4.1 Model prediction accuracy

Our investigation into the predictive capabilities of various machine learning techniques for Chinese stock market returns is presented in Table 1. Results show the monthly out-of-sample *R*^2^ of the Factor-GAN model alongside comparative models, including linear regression (LR), elastic network (Enet), random forest (RF), neural networks (NN), and LSTM.

**Table 1:** Monthly out-of-sample prediction performance (in percentage %).

	LR	Enet	RF	NN	LSTM	FactorGAN
$R_{\text{os}}^{2}$	-4.26	0.11	0.24	0.30	0.57	1.12
RMSE	15.65	15.10	14.59	14.21	14.04	13.93

The widely used LR method in economics and statistics exhibits the least favorable performance, recording a monthly out-of-sample $R_{\text{os}}^{2}$ of -4.26. This outcome aligns with our expectations, as linear regression’s absence of regularization mechanisms renders it susceptible to overfitting on in-sample data, limiting its ability to generalize to new datasets. In contrast, the Enet model, specifically engineered to mitigate issues like multi-collinearity and overfitting, demonstrates a marked improvement over the traditional linear approach, achieving an R2os of 0.11. The Enet model’s advantage is attributed to its integration of L1 and L2 regularization techniques, which not only reduce the model’s complexity but also enhance its capability to manage datasets with numerous feature variables, thereby demonstrating the power of dimensionality reduction.

Machine learning methods distinguish themselves by constructing complex nonlinear functions, enabling a thorough extraction and analysis of the intricate interactions between factors. The RF and NN models, in particular, showcase notable advancements in predictive performance compared to both LR and ENET, with out-of-sample R2 values of 0.24 and 0.30, respectively. Their success can be attributed to their ability to model nonlinear relationships, making them more adaptable to the multifaceted nature of financial data.

Deep learning models, especially LSTM, excel remarkably, achieving an $R_{\text{os}}^{2}$ of 0.57. LSTM’s memory cells are adept at capturing and leveraging early time-series information, which is pivotal in forecasting contexts where past trends and patterns significantly influence future outcomes. Furthermore, Factor-GAN, with its innovative incorporation of a dynamic game mechanism between the generator and discriminator networks, emerges as a standout performer. Its predictive accuracy, nearly double that of LSTM, is a testament to the strength of combining generative adversarial learning with factor analysis, significantly exceeding other control models.

When evaluating model performance through Root Mean Square Error (RMSE) metric, LR records the highest forecast error at 15.65, followed by Enet with an error of 15.10. The more advanced machine learning approaches, including tree models and neural networks, achieve lower RMSE values, all below 15%, reflecting their enhanced predictive accuracy. Notably, Factor-GAN boasts the smallest forecast error at merely 13.93, highlighting its exceptional ability to minimize discrepancies between actual and predicted values.

**Fig 6:**
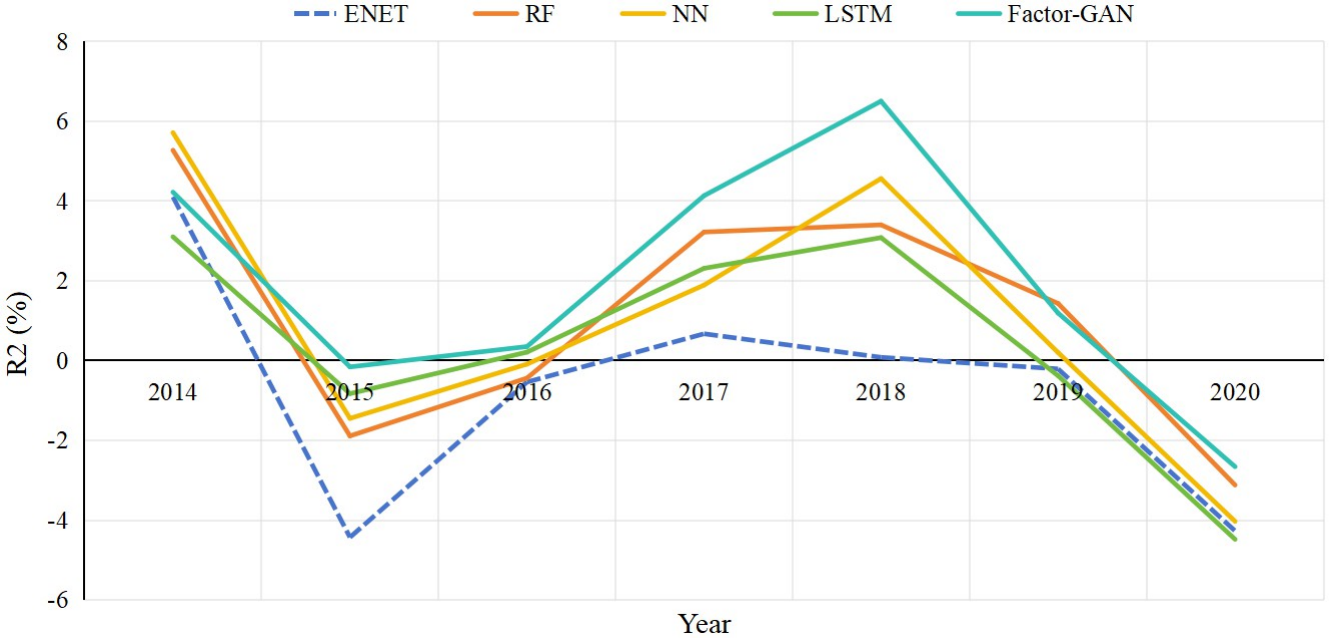
Annual out-of-sample prediction fluctuation.

Fig 6 visually depicts the annual out-of-sample *R*^2^ values for each model from January 2014 to December 2020. Compared to linear and machine learning models, Factor-GAN demonstrates superior stability across different years. During the initial stage from 2014 to 2016, the Enet model’s $R_{\text{os}}^{2}$ drops to its lowest point at -4.43%, with a volatility of 8.58%. In contrast, Factor-GAN exhibits remarkable stability throughout the entire testing period, with a volatility of only 4.38%. Notably, this stability is particularly evident during the 2015 Chinese stock market crash. Subsequently, Factor-GAN consistently outperforms the control models in prediction accuracy, peaking around 2018 with an impressive 6.50%. However, from 2019 to 2020, the COVID-19 pandemic causes increased stock market volatility and a decline in prediction accuracy across all methods.

The observed comparative performance among all methods closely aligns with their monthly outcomes, but the annual $R_{\text{os}}^{2}$ values are nearly an order of magnitude larger. Our findings are in line with Gu et al.’s study on the U.S. market [38], demonstrating that machine learning models can detect long-lasting risk premiums persisting across various business cycles, rather than solely capturing temporary market inefficiencies.

### 4.2 Portfolio performance

We delve into the analysis of portfolio performance, constructing portfolios based on the returns generated by the Factor-GAN model. Specifically, each month, portfolios are created based on predicted returns of individual stocks by sorting them in ascending order. The top 10 stocks with the smallest predicted returns form Group L, while the top 10 stocks with the highest predicted returns constitute Group H. Long-short hedging portfolios, denoted as H-L, are then created by going long on Group H and shorting Group L. These portfolios are held for one month, and the process is repeated monthly until the end of the sample period. Portfolio returns are weighted by the outstanding market value of each stock.

Table 2 displays the performance of portfolios constructed by each model, featuring four commonly used financial indicators: average predicted monthly return (Avg), standard deviation of monthly returns (Std), Sharpe ratio (SR), and maximum drawdown of the portfolios (MaxDD).

**Table 2:** Performance measures of long-short portfolios.

Portfolio	Indicator	LR	Enet	RF
H-L	Avg (%)	-0.03	0.77	1.12
	Std (%)	6.55	6.04	5.45
	SR	-0.02	0.44	0.71
	MaxDD (%)	25.08	19.72	13.06
**Portfolio**	**Indicator**	**NN**	**LSTM**	**FactorGAN**
H-L	Avg (%)	1.23	1.57	1.96
	Std (%)	5.68	5.43	5.25
	SR	0.75	1.00	1.29
	MaxDD (%)	10.65	6.43	9.77

The results illustrate the exceptional performance of the H-L portfolios constructed using the Factor-GAN model. These portfolios achieved an impressive average monthly return of 1.96%, equivalent to an annualized return of 23.52%, outperforming the LSTM by a substantial margin of approximately 25%. Moreover, the GAN-based portfolio excelled in terms of risk-adjusted performance, boasting the highest Sharpe ratio of 1.29, underscoring its exceptional risk-return trade-off. Additionally, it exhibits resilience against market fluctuations, registering the second-lowest maximum drawdown of 9.77. These findings suggest the superiority of the Factor-GAN approach in enhancing portfolio profitability and risk management.

It’s noteworthy that both the LSTM and NN-based portfolios show significant performance improvements compared to traditional models like LR and Enet. The results underscore the overall advantage of employing machine learning techniques in portfolio forecasting, as these models effectively capture complex patterns and interactions within financial data, leading to enhanced investment outcomes.

**Fig 7:**
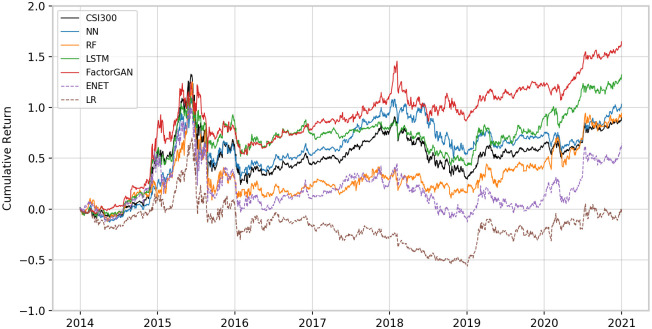
Cumulative return of the long-short portfolio during testing period.

Fig 7 represents the cumulative returns evolution for the long-short portfolios, with the CSI300 index serving as a reference benchmark. Throughout the back-testing period, Factor-GAN (red line) consistently outperforms its counterparts, demonstrating remarkable cumulative gains of 164.77%. The LSTM (green line) and the NN (blue line), both falling under the category of neural network models, exhibited relatively similar performances. Meanwhile, the tree model’s performance lagged behind.

The performance of all machine learning portfolios proves superior to the penalized linear model. Among them, the cumulative returns of portfolios based on neural networks outperform the contemporaneous CSI300 index. Even during the global economic shock resulting from the COVID-19 pandemic, these portfolios remain resilient, with no substantial downturn observed at the portfolio level.

### 4.3 Which predictors are important

Understanding the economic mechanisms underlying machine learning models is crucial, given their often enigmatic “black box” nature. In our comprehensive study, where numerous factors potentially influence future stock returns, our aim is to identify the important predictors within the model. Initially, we regress the monthly returns of the H-L portfolio using the Fama-French three-factor (FF3) and five-factor (FF5) models.

In Table 3, FF3−*a* and FF5−*a* represent the excess returns of the multi-factor model, while the T-value corresponds to the t-statistic. Notably, FF3−*a* and FF5−*a* of the Enet-based portfolios are statistically insignificant at the 10% level, suggesting that the excess returns in the penalized linear model can be explained by the multi-factor model. In contrast, portfolios generated by machine learning methods, especially the Factor-GAN model, exhibit statistical significance at the 5% level, with monthly excess returns reaching 0.88 and 0.86, respectively. These results underscore that portfolio returns constructed through machine learning defy explanation by traditional economic models.

**Table 3:** Statistics of portfolios in FF3 and FF5 models.

	Enet	RF	NN	LSTM	FactorGAN
FF3-a(%)	0.36	0.31	0.58	0.67	0.88
T-value	1.14	8.75	12.36	16.21	18.78
FF5-a(%)	0.15	0.30	0.57	0.65	0.86
T-value	1.25	9.07	13.62	15.45	18.14

**Fig 8:**
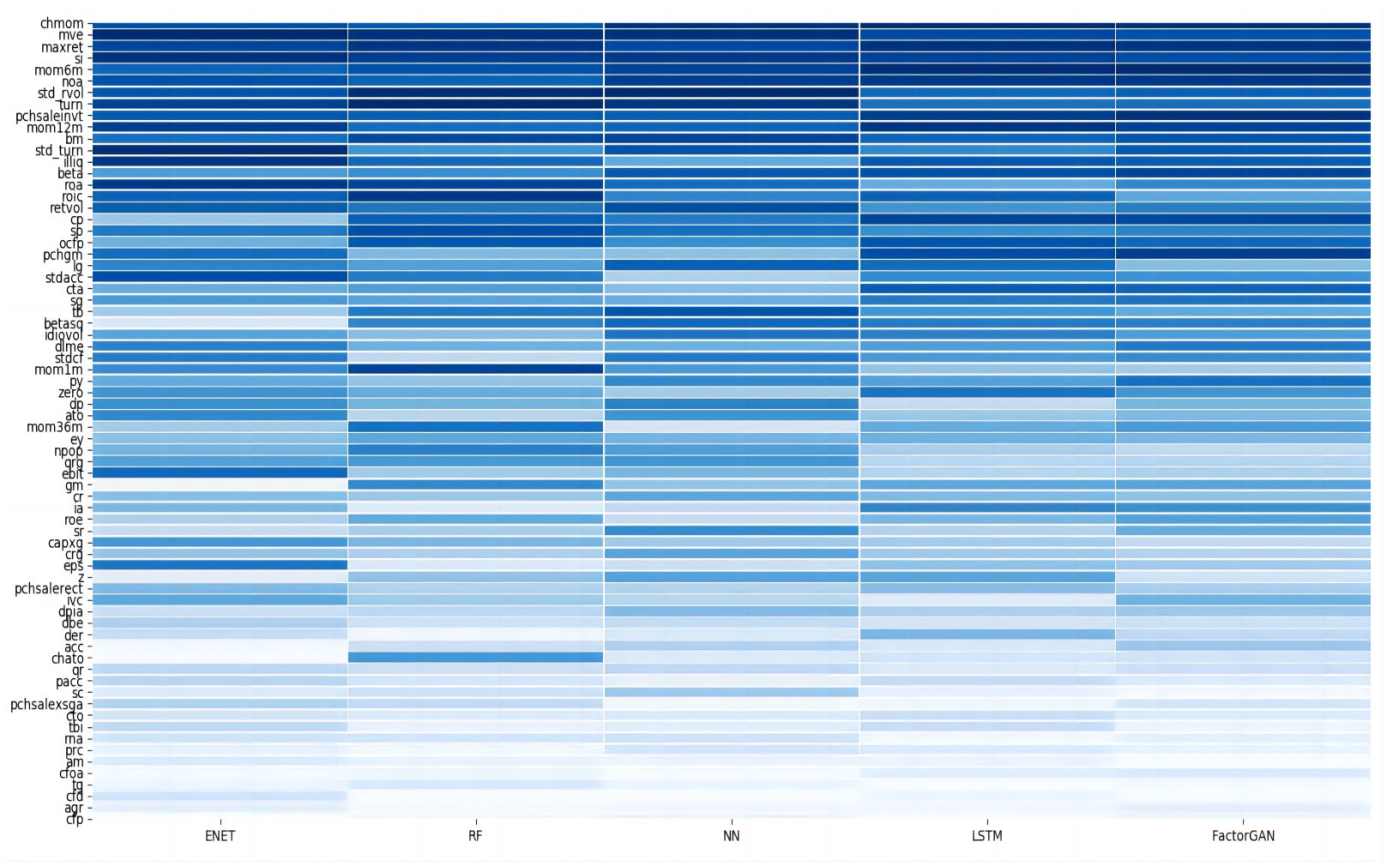
The variable importance of factors in various machine learning models.

Subsequently, we investigate which among the 70 firm-level characteristics plays a key role in predicting stock returns for each model. For a specific model, we calculate the reduction in $R_{\text{os}}^{2}$ when setting values of a particular predictor to zero within each training sample, and average them into a single measure of variable importrnce for each predictor. Further, we standardize these importance values to ensure they sum up to one, allowing for an easier comparison of predictors. The results show that not all firm characteristics are equally significant in predicting stock returns, and their importance varies among prediction models. Fig 8 provides an overview of the influence of firm factors on returns in the context of ML models. The vertical axis represents the 70 characteristics, ranked from most influential (top) to least influential (bottom), based on their contribution across all models. The horizontal axis corresponds to the different ML algorithms, while the color gradient indicates the variable importance of predictors, with darker colors signifying a stronger influence and lighter colors suggesting less impact.

Fig 8 reveals that the most crucial predictors can be categorized into three groups. The first group encompasses factors related to stock price trends, including intermediate momentum indicators (*mom*6, *mom*12), maximum daily returns (*maxret*), and changes in momentum (*chmom*). This observation aligns with the principles of behavioral finance, suggesting that the behavioral biases of irrational investors lead to stock price mispricing, resulting in excess returns for momentum-related factors.

The second group comprises trading friction indicators, such as market liquidity (*turn*, *illiq*), market volatility (*std*_*turn*, *std*_*rvol*), and market capitalization (*mve*). Turnover rate and its variance rank prominently within the neural network models in this category. A higher turnover rate indicates more active stock trading and increased liquidity, making these stocks more attractive to investors. However, it’s essential to note that a sudden amplification in turnover variance often accompanies higher volatility risk.

The third group includes fundamental indicators, such as net operating assets (*noa*), book-to-market equity (*bm*), the difference between sales growth and inventory growth (*pchsaleinvt*), and the sales-to-inventory ratio (*si*). It’s noteworthy that *noa* and *bm* belong to operating performance indicators, assessing a company’s resource utilization efficiency and management ability. On the other hand, *pchsaleinvt* and *si* reflect a company’s turnover capability. Exceptional companies in the industry can rapidly liquidate their products, which enhances their resilience to risks and lowers overall business risk. In summary, these fundamental predictors emerge as influential drivers of excess returns in stock investments.

Our research not only confirms but also unveils notable distinctions from previous studies. For instance, Chen et al. [51] leveraged deep learning techniques to estimate an asset pricing model for individual stock returns. They highlighted the significance of several fundamental characteristics, such as size, value, investment, and profitability attributes, notably the dividend-price ratio and book-to-market ratio. These outcomes mirror our observations in the Chinese market, reinforcing the vital role of fundamental predictors in the model. Gu et al. [38], employing machine learning methods to forecast the U.S. market, emphasized recent price trends and liquidity variables as the most influential categories. Conversely, Leippold et al. [56] suggested that the momentum predictors carried minimal significance in the Chinese stock market, except for the maximum recent returns (*maxret*). Yet, our study reveals that medium- and long-term momentum emerges as a crucial predictor in deep learning models, such as LSTM and Factor-GAN, and other momentum attributes like *mom*1*m* and *chmom* also exhibit an influence on excess returns.

Furthermore, our research highlights variations among different machine learning techniques regarding predictor importance. The random forest places a strong emphasis on liquidity indicators such as *std*_*turn*, *std*_*rvol*, and *illiq*. This emphasis may be attributed to RF models selecting a subset of stock characteristics when constructing decision trees. Therefore, these predictors can become pivotal in certain decision trees and then have an impact on the whole tree model. In contrast, deep learning algorithms like LSTM and Factor-GAN display a distinct preference for momentum and fundamental predictors. This preference may be linked to the unique structure of the memory unit, which can retain and quickly forget information, allowing fundamental financial predictors like *noa*, *si*, and others to influence stock return.

## 5 Subsample analysis

Our previous research has demonstrated the GAN model’s remarkable superiority in predictive capabilities when compared to other machine learning methods. To further validate our findings and gain deeper insights into the underlying characteristics driving performance, we make a comprehensive examination of the key predictors.

The Chinese stock market stands out with its unique characteristics, setting it apart from its U.S. counterpart. These distinct features encompass a significant presence of state-owned enterprises, diverse companies listed on both the A- and H-shares markets, and an inclination among investors for small-cap stocks with growth potential. Given these disparities, we conduct two sets of subsample tests to dissect variations in the prediction accuracy and ascertain the relative variable importance in the context of the Factor-GAN model.

### 5.1 State ownership

State-owned enterprises, where the government plays a pivotal role in appointing managers and overseeing performance, hold a crucial position in China’s capital market. As of the end of 2020, the number of SOEs had exceeded 460,000, constituting 40.9% of the total assets of non-financial enterprises in China. Unlike the markets in Europe and the U.S., where private enterprises dominate, SOEs are the cornerstone of the Chinese economy, forming an extensive and dominant asset-scale system that extends its influence across nearly every industrial sector. However, government involvement in investment decision-making raises concerns regarding their financial performance [59]. Research by Wei et al. [60] reveals that SOEs tend to be less profitable and productive compared to privately-owned firms. This is attributed to more lenient monitoring, softer budget constraints, and comparatively weaker employees and management. These factors hinder the overall performance of SOEs and contribute to their differentiation from non-SOEs. Therefore, it becomes essential to assess the impact of the notably high proportion of SOEs in the market on stock return forecasts.

In this study, we divide the stocks into two distinct subgroups: SOEs, totaling 1,090 instances, and non-SOEs, totaling 2,529 instances. Our primary objective is to evaluate the price prediction accuracy of the Factor-GAN model within these subgroups. Table 4 clearly highlights a substantial performance gap.

**Table 4:** Prediction accuracy of the Factor-GAN model on subsamples.

Subsamples	R2(%)	RMSE(%)
SOE	1.43	15.22
non-SOE	1.08	14.76
Dual Listing	1.37	16.28
A-share Listing	1.15	14.01

The RMSE value for SOEs is higher compared to non-SOEs, indicating a less accurate prediction. Specifically, the difference in $R_{\text{os}}^{2}$ is particularly striking, with SOEs achieving 1.43 compared to 1.08 for non-SOEs, leading to a decrease of about 30%. This suggests that the Factor-GAN model is more adept at capturing the stock return patterns of SOEs, resulting in a more accurate representation of their behaviors.

**Fig 9:**
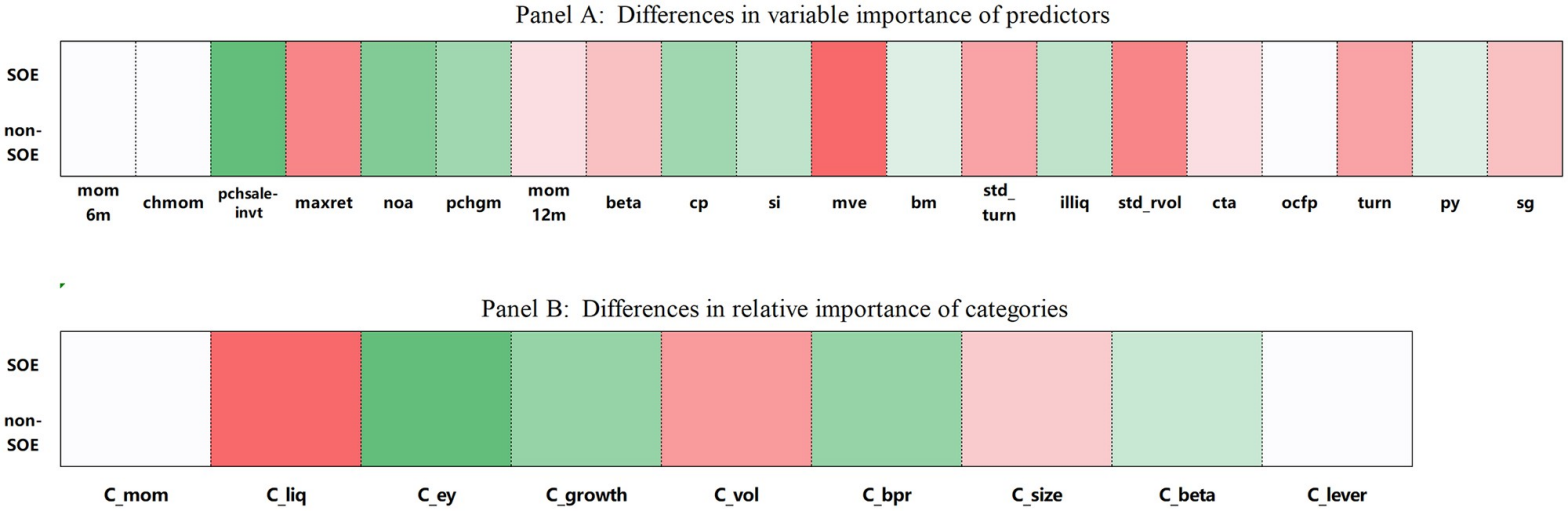
Relative variable importance of SOEs and non-SOEs.

In order to unveil the black box of machine learning and trace the economic mechanism behind it, we further explore which firm-level predictors cause differences in subsamples. Fig 9 illustrates the difference in relative variable importance between SOEs and non-SOEs.

Panel A of Fig 9 depicts the variations in the importance of the top 20 influential predictors in the entire sample. The red gradient signifies an increase in variable importance, while the green gradient indicates a decrease, with darker colors signifying more significant changes. As we transition from SOEs to non-SOEs, we note that momentum predictors, which play a pivotal role in the Factor-GAN model, exhibit a remarkable degree of stability. Specifically, these include: (1) *mom*6*m*, representing 6-month momentum, denoting the cumulative daily return from six months prior, serving as a medium-term momentum indicator. (2) *chmom*, indicating the change in 6-month momentum, representing momentum reversals in the medium term.

In contrast, the variable importance of fundamental characteristics experiences a significant decline. Predictors such as *pchsaleinvt* and *pchgm*, which gauge the turnover ability of enterprises, as well as *noa* and *cp*, representing the operating ability of enterprises, see a marked reduction in their influence. Particularly noteworthy is the role of *maxret*. As suggested by Bali et al. [61], investors show preferences for assets with lottery-like payoffs, and many investors exhibit a lack of diversification. Cross-sectional regressions indicate a negative relationship between the maximum daily return and expected stock returns, with this effect being more pronounced in small-cap stocks. These findings corroborate our hypothesis that retail investors significantly impact the stock price volatility of non-SOEs (small-cap stocks), aligning with the GAN model’s emphasis on *maxret*.

Rather than further exploring characteristics at the bottom of the rankings, we have organized these attributes into representative categories. By examining the overall changes in category importance, we aim to mitigate potential data outliers. The experimental results are displayed in Panel B of Fig 9.

Interestingly, for both SOEs and non-SOEs, momentum indicators emerge as the primary drivers of earnings predictability. This differs somewhat from the findings of Leippold et al. [56], who utilized shallow neural networks to model stock returns and concluded that the divergence in return predictions was due to an overemphasis on the momentum factor for non-SOEs. Furthermore, during the transition from SOEs to non-SOEs, there is a notable increase in the importance of liquidity (*C*_*liq*) and volatility (*C*_*vol*) metrics, while the significance of earning (*C*_*ey*) and valuation (*C*_*bpr*) metrics undergoes a substantial decrease. This change can be attributed to the training process of the Factor-GAN model, where the generator and discriminator engage in adversarial learning until the generated samples are realistic enough that the discriminator cannot differentiate between real and predicted data. The competitive process aids in uncovering the role of fundamental factors with a substantial impact on stock returns, allowing it to discern differences between subsamples. Overall, these shifts offer a glimpse into how different types of companies, with varying ownership structures and operating environments, prioritize and respond to different factors influencing their stock returns.

### 5.2 Dual listing

Established around 1990, China’s stock market features a unique dual-listing system [62]. In this study, dual-listed stocks refer to companies simultaneously listed on China’s A-share market and the Hong Kong Stock Exchange (H-share). Notably, A-shares and H-shares exhibit distinct characteristics, leading to significant differences in their valuation. A-share markets primarily cater to domestic Chinese investors, often dominated by retail investors who may lack extensive financial knowledge and expertise. Additionally, these markets frequently witness herd behavior, particularly during bullish market phases [63]. This propensity for collective decision-making can lead to overvaluation, where stock prices surpass their fundamental values. In contrast, Hong Kong’s capital market is renowned for its maturity, openness, and accessibility to institutional and foreign investors [64]. This enhanced institutional presence and international participation create a more balanced and diversified investor base.

**Fig 10:**
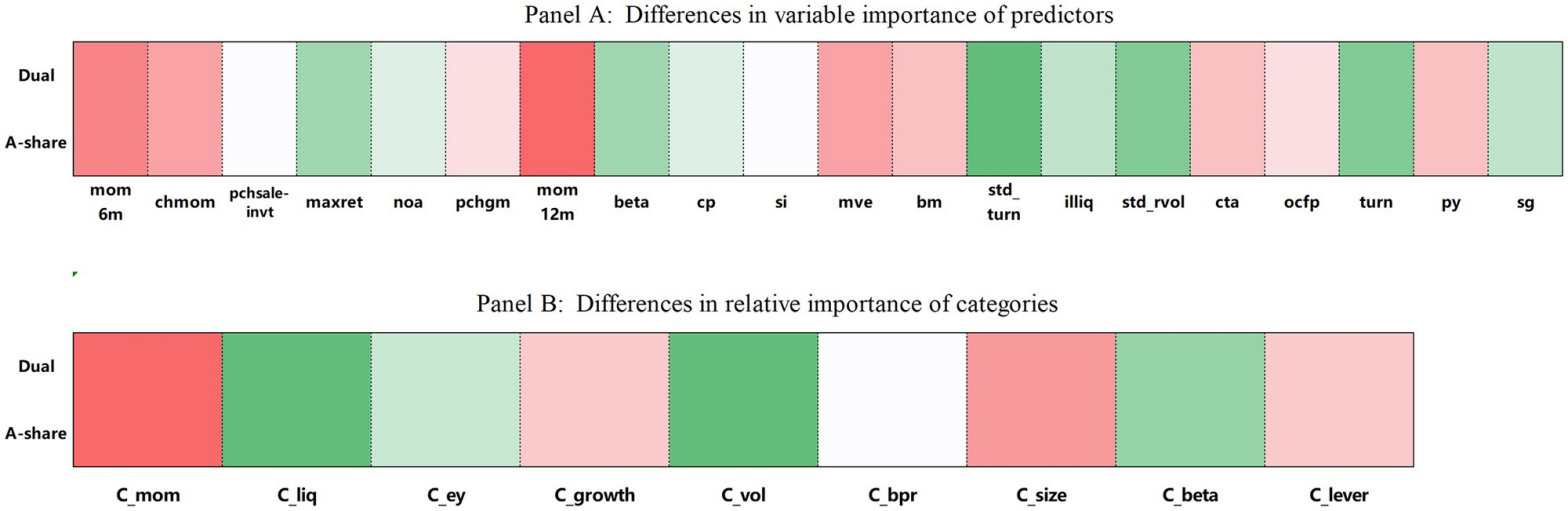
Relative variable importance of dual-listed and A-share listed companies.

In our analysis, we divide the samples into two distinct groups: dual-listed companies and those exclusively listed on the A-share market. The third and fourth rows of Table 4 reveal the predictive accuracy of stock returns using the Factor-GAN model. For dual-listed stocks, we observe an out-of-sample *R*^2^ of 1.37, which surpasses that of companies with unilateral listings by 19.13%. Additionally, the RMSE value for dual-listed stocks reaches 16.28. Fig 10 further illustrates the variable importance in the context of dual-listing.

As depicted in Panel A of Fig 10, dual-listed firms exhibit a distinct emphasis on liquidity predictors, such as *std*_*rvol*, *turn*, and *std*_*turn*. These findings corroborate the insights of prior research. For instance, Foerster and Karolyi [65] employed an indirect measurement method to examine the relationship between enhanced stock liquidity through dual-listing and the resulting increase in corporate value. Their research, which employed listing location as an indicator of liquidity, demonstrated significant variations in abnormal returns generated by dual listings depending on the listing location. Likewise, Kot and Tan [66] investigated the impacts of dual-listing on stock price informativeness and liquidity. Their study revealed that the issuance of A-shares has a positive effect and tends to boost the turnover of H-shares. This potential spillover effect on liquidity may arise from increased visibility resulting from A-shares issuance, a broader investor base, and an expanded market capitalization.

Conversely, companies listed on the A-share market demonstrate a pronounced reliance on indicators predicting price trends, with a special focus on medium-term momentum indicators like *mom*6*m* and *mom*12*m*. This tendency aligns with observations made by Li et al. [67], who note that investors in the A-share market are characterized by a significant representative bias. This bias leads them to disproportionately value short-term data at the expense of longer-term insights, often resulting from a constrained capacity for comprehensive data analysis. Furthermore, these investors frequently exhibit herd behavior, basing their investment choices on recent returns. This inclines them to follow prevailing market trends, joining in on upward movements and shying away from downturns. This behavior underscores a reactive investment strategy, influenced heavily by recent market performance and the actions of others, rather than a balanced assessment of potential long-term value and risks.

We then delve into the assessment of the relative importance of categories within dual-listed and A-listed stocks, as illustrated in Panel B of Fig 10. Notably, during the shift from dual listings to A-share listings, several categories experience a significant increase in their relative importance. Foremost among them is the momentum category (*C*_*mom*), followed by market capitalization (*C*_*size*) and growth (*C*_*growth*). Examining the underlying economic reasons for these shifts, it becomes apparent that A-share markets witness a relatively high proportion of retail investors. As Ng and Wu [68] argued, Chinese participants often exhibit a strong preference for investing in small-cap stocks, particularly those associated with growth. This preference creates a surge in interest, driving the importance of the momentum and growth categories. The dual-listing mechanism offers companies an opportunity to gain exposure in diverse markets, enhance their international visibility, bolster their reputation, and expand their financing channels. Consequently, investors exhibit a heightened concern for factors such as transaction friction in these markets, contributing to the fluctuations in category importance.

## 6 Conclusion and future work

### 6.1 Conclusion

In this study, leveraging data spanning from 2002 to 2020 of all stocks in China’s A-share market, we construct a comprehensive factor database comprising 70 firm-level characteristics. Combining multi-factor models with deep neural networks, our deep learning-based multi-factor pricing model proves instrumental in predicting stock market returns and formulating factor investment strategies. The key findings are as follows:

Firstly, our study finds that deep learning methods, in comparison to linear models and shallow neural networks, significantly improve stock prediction accuracy. The Factor-GAN model, in particular, demonstrates effectiveness, achieving a monthly out-of-sample *R*^2^ of 1.12% and a minimal prediction error of 13.93%. Moreover, Factor-GAN exhibits more consistent performance across various market conditions when compared to other nonlinear models, showcasing its robustness and stability throughout the testing period.

Secondly, portfolio construction based on model predictions reveals that the long-short portfolio with GANs strategy outperforms others. The Factor-GAN portfolio achieve a remarkable monthly return of 1.96% (23.52% annualized) with a Sharpe ratio of 1.29, boasting the highest cumulative return during the backtesting period at 164.77%. These results underscore the superior performance of Factor-GAN.

Thirdly, we investigate the economic mechanisms driven by deep learning at the factor level. Experimental findings reveal that the influential predictors within the Factor-GAN model can be categorized into three groups: price trend, trading friction, and fundamental indicators. Considering the unique characteristics of the Chinese market, a subsample analysis is conducted. Results indicate that during the transition from SOEs to non-SOEs, the variable importance of fundamental factors experiences a significant reduction, making way for liquidity and volatility indicators. Additionally, in comparison to dual-listed companies, A-share listed entities exhibit a greater emphasis on momentum and growth indicators while allocating less focus to liquidity.

### 6.2 Implication and future work

The theoretical contribution of this study lies in its expansion of the literature on empirical asset pricing and explainable artificial intelligence. By leveraging machine learning technology, this study offers novel insights into asset pricing research, extending the application of asset pricing theory to factor investment strategies. Specifically, we integrate deep learning into multi-factor models to unveil the intricate operational dynamics of financial markets, thereby reshaping traditional financial research paradigms. Additionally, we endeavor to explain the economic theoretical mechanisms underlying deep learning. The Factor-GAN framework embodies principles of explainability by clarifying the dynamic interactions and significance of various market factors in stock price prediction. Our findings advocate for the development of AI models that not only excel in performance but also offer transparency into their decision-making processes. This approach fosters trust and understanding among users, which is particularly vital in finance, where the rationale behind predictions and investment decisions is as crucial as their outcomes.

The practical significance lies in offering valuable insights for participants in the financial market. Investors can leverage enhanced prediction accuracy and optimized portfolios facilitated by deep learning models, empowering them to make more informed investment decisions and construct portfolios with improved risk-adjusted returns. Financial analysts can enrich their analytical toolkit by integrating deep learning techniques, especially by harnessing the Factor-GAN model’s emphasis on various factors. This enables analysts to better understand stock market dynamics and focus on key driving factors. Regulators, too, can benefit by utilizing deep learning models for more effective market trend monitoring and anomaly detection. Recognizing the variable importance of factors, as emphasized in this study, enables regulators to formulate targeted policies, thereby enhancing the quality of financial market operations.

When applying the Factor-GAN framework in practice, several considerations should be addressed to harness its potential effectively. Foremost is the emphasis on data preparation and quality assurance. The accuracy of input data directly influence Factor-GAN’s performance. A diverse dataset, covering a broad spectrum of market factors, is essential for generating reliable predictions. Secondly, customization and calibration play a crucial role. Factor-GAN’s parameters, including factor selection and model weighting, must be carefully adjusted to align with specific market contexts and investment objectives. Lastly, given the financial market’s inherent volatility, continuous monitoring and adjustment is indispensable.

While acknowledging the limitations of this study, it suggests promising avenues for future research in several aspects. Firstly, the focus on the emerging Chinese market makes the findings specific to these unique conditions. Researchers are encouraged to apply the proposed approach to other financial markets, conducting comparative analyses to assess performance and establish the model’s generalizability. Comparative studies across diverse financial markets can validate the broader applicability of the proposed model.

Secondly, the factor database in this study exclusively incorporates firm-level characteristics, excluding macro factors. Future research endeavors should explore the interplay between macro and micro factors and their combined impact on stock prediction. Expanding the scope to include macroeconomic influences holds the potential to offer a more comprehensive understanding of the dynamics driving stock market predictions, paving the way for more robust and holistic asset pricing models.

Thirdly, Factor-GAN currently integrates generative adversarial networks solely with the multi-factor pricing model. Although widely employed in financial research, the Fama-French factor model exhibits inherent limitations. In forthcoming investigations, we intend to enhance our methodology by integrating generalized linear models, such as LASSO and Ridge regression, in conjunction with deep learning techniques. By broadening the scope of model integration, we aspire to overcome the inherent limitations of current methodologies and deliver more robust insights into factor investing dynamics.

Moreover, while Factor-GAN framework showcases promising outcomes, it is crucial to undertake further comparisons with sophisticated time-series models, including RNN, GRU, Transformer, TCN, and BERT, to thoroughly affirm its distinguished performance. This comparative analysis will constitute a significant aspect of our forthcoming research endeavors, aimed at providing an exhaustive assessment of Factor-GAN’s proficiency in stock price forecasting and factor investment strategies.

## Supporting information

S1 Appendix Details on micro-firm characteristics. (DOCX)
