# Supplementary material for: Factor-GAN: Enhancing stock price prediction and factor investment with Generative Adversarial Networks
Source: PLoS One. 2024 Jun 25;19(6):e0306094. doi: 10.1371/journal.pone.0306094 (PMC11198854; doi:10.1371/journal.pone.0306094)
Supplement: S1 Appendix — (DOCX) [file pone.0306094.s001.docx]

**Supporting information**

S1 Code and dataset

URL: https://figshare.com/articles/dataset/Factor-GAN/25699419

DOI: 10.6084/m9.figshare.25699419

S1 Appendix.

**Table1. Details on micro-firm characteristics.**

| No. | Acronym | Stock Characteristics | Frequency | Category |
| --- | --- | --- | --- | --- |
| 1 | acc | accruals | Quarterly | ey |
| 2 | agr | Asset growth | Quarterly | growth |
| 3 | am | assets-to-market | Quarterly | bpr |
| 4 | ato | asset turnover | Quarterly | ey |
| 5 | beta | market Beta | Monthly | beta |
| 6 | betasq | Beta squared | Monthly | beta |
| 7 | bm | book-to-market equity | Quarterly | bpr |
| 8 | capxg | capital expenditure growth | Quarterly | growth |
| 9 | cfd | cash flow-to-debt | Quarterly | lever |
| 10 | cfoa | cash flow over assets | Quarterly | ey |
| 11 | cfp | cash flow-to-price | Quarterly | bpr |
| 12 | chato | Change in asset turnover | Quarterly | liq |
| 13 | chmom | change in 6-month momentum | Monthly | mom |
| 14 | cp | cash productivity | Quarterly | bpr |
| 15 | cr | current ratio | Quarterly | lever |
| 16 | crg | current ratio growth) | Quarterly | lever |
| 17 | cta | cash-to-assets | Quarterly | ey |
| 18 | cto | capital turnover | Quarterly | ey |
| 19 | dbe | change in shareholders’equity | Quarterly | ey |
| 20 | der | debt-to-equity ratio | Quarterly | lever |
| 21 | dlme | long term debt-to-market equity | Quarterly | lever |
| 22 | dp | dividend-to-price ratio | Quarterly | ey |
| 23 | dpia | changes in PPE and inventory-to-assets | Quarterly | bpr |
| 24 | ebit | Earnings before interests and taxes | Quarterly | ey |
| 25 | eps | Earning PerShare | Quarterly | bpr |
| 26 | ey | earnings yield | Quarterly | ey |
| 27 | gm | gross margins | Quarterly | ey |
| 28 | ia | investment-to-assets | Quarterly | ey |
| 29 | idiovol | idiosyncratic return volatility | Monthly | vol |
| 30 | illiq | illiquidity | Monthly | liq |
| 31 | ivc | inventory change | Quarterly | size |
| 32 | lg | liability growth | Quarterly | lever |
| 33 | maxret | Maximum daily return | Monthly | mom |
| 34 | mom1m | 1-month momentum | Monthly | mom |
| 35 | mom6m | 6-month momentum | Monthly | mom |
| 36 | mom12m | 12-month momentum | Monthly | mom |
| 37 | mom36m | 36-month momentum | Monthly | mom |
| 38 | mve | size | Monthly | size |
| 39 | noa | net operating assets | Quarterly | ey |
| 40 | npop | net payout over profits | Quarterly | ey |
| 41 | ocfp | operating cash flow-to-price | Quarterly | bpr |
| 42 | pacc | percent accruals | Quarterly | bpr |
| 43 | pchgm | % change in gross margin - % change in sales | Quarterly | growth |
| 44 | pchsaleinvt | % change in sales - % change in inventory | Quarterly | growth |
| 45 | pchsalerect | % change in sales - % change in A/R | Quarterly | ey |
| 46 | pchsalexsga | % change in sales - % change in SG&A | Quarterly | growth |
| 47 | prc | price | Monthly | liq |
| 48 | py | payout yield | Quarterly | bpr |
| 49 | qr | quick ratio | Quarterly | lever |
| 50 | qrg | quick ratio growth | Quarterly | lever |
| 51 | retvol | return volatility | Monthly | vol |
| 52 | rna | return on net operating assets | Quarterly | ey |
| 53 | roa | return on assets | Quarterly | ey |
| 54 | roe | return on equity | Quarterly | ey |
| 55 | roic | return on invested capital | Quarterly | ey |
| 56 | sc | sales-to-cash | Quarterly | ey |
| 57 | sg | sustainable growth | Quarterly | growth |
| 58 | si | sales-to-inventory | Quarterly | bpr |
| 59 | sp | sales-to-price | Quarterly | bpr |
| 60 | sr | Sales growth | Quarterly | growth |
| 61 | std_rvol | volatility of ＲMB trading volume | Monthly | liq |
| 62 | std_turn | volatility of turnover | Monthly | liq |
| 63 | stdacc | Accrual volatility | Quarterly | ey |
| 64 | stdcf | Cash flow volatility | Quarterly | ey |
| 65 | tb | Debt capacity/firm tangibility | Quarterly | lever |
| 66 | tbi | taxable income-to-book income | Quarterly | ey |
| 67 | tg | tax growth | Quarterly | bpr |
| 68 | turn | share turnover | Monthly | liq |
| 69 | z | Z-score | Quarterly | ey |
| 70 | zero | zero trading days | Monthly | liq |

**Table 2. Statistical descriptions of firm-level factors.**

| Factor | Mean | Std | No. | Factor | Mean | Std |
| --- | --- | --- | --- | --- | --- | --- |
| acc | 0.033 | 0.817 | 36 | mom12m | 0.249 | 0.593 |
| agr | 0.038 | 0.852 | 37 | mom36m | 0.666 | 0.978 |
| am | -0.040 | 0.520 | 38 | mve | -0.004 | 0.803 |
| ato | 0.056 | 1.004 | 39 | noa | 0.065 | 0.542 |
| beta | -0.012 | 0.926 | 40 | npop | 0.002 | 0.914 |
| betasq | -0.018 | 0.877 | 41 | ocfp | -0.003 | 0.786 |
| bm | 0.025 | 0.834 | 42 | pacc | -0.001 | 0.991 |
| capxg | 0.026 | 1.045 | 43 | pchgm | 0.048 | 0.892 |
| cfd | 0.075 | 0.932 | 44 | pchsaleinvt | 0.063 | 0.887 |
| cfoa | 0.060 | 0.726 | 45 | pchsalerect | 0.048 | 0.968 |
| cfp | 0.037 | 0.978 | 46 | pchsalexsga | 0.058 | 0.924 |
| chato | 0.056 | 0.962 | 47 | prc | -0.033 | 0.953 |
| chmom | 0.005 | 0.880 | 48 | py | -0.004 | 0.813 |
| cp | -0.020 | 0.318 | 49 | qr | 0.018 | 0.907 |
| cr | 0.015 | 0.987 | 50 | qrg | 0.000 | 0.868 |
| crg | -0.001 | 0.856 | 51 | retvol | 0.057 | 0.936 |
| cta | 0.046 | 0.974 | 52 | rna | -0.002 | 0.912 |
| cto | 0.029 | 0.978 | 53 | roa | 0.001 | 1.005 |
| dbe | 0.049 | 0.841 | 54 | roe | 0.004 | 1.001 |
| der | -0.052 | 0.415 | 55 | roic | -0.003 | 0.875 |
| dlme | 0.014 | 0.998 | 56 | sc | -0.034 | 0.692 |
| dp | 0.022 | 1.007 | 57 | sg | 0.027 | 0.999 |
| dpia | 0.066 | 0.826 | 58 | si | 0.001 | 1.004 |
| ebit | 0.003 | 0.900 | 59 | sp | 0.013 | 0.983 |
| eps | 0.073 | 0.609 | 60 | sr | 0.004 | 0.944 |
| ey | 0.104 | 0.788 | 61 | std_rvol | -0.024 | 0.918 |
| gm | 0.048 | 0.895 | 62 | std_turn | 0.008 | 1.007 |
| ia | 0.031 | 0.904 | 63 | stdacc | 0.004 | 0.925 |
| idiovol | -0.050 | 0.897 | 64 | stdcf | 0.013 | 0.980 |
| illiq | -0.055 | 0.634 | 65 | tb | 0.020 | 0.966 |
| ivc | 0.043 | 0.994 | 66 | tbi | 0.020 | 1.013 |
| lg | 0.007 | 0.846 | 67 | tg | 0.010 | 0.998 |
| maxret | -0.041 | 0.357 | 68 | turn | 0.014 | 1.095 |
| mom1m | 0.026 | 0.149 | 69 | z | 0.030 | 1.066 |
| mom6m | 0.113 | 0.355 | 70 | zero | -0.015 | 0.820 |
